# Marital status and survival in patients with renal cell carcinoma

**DOI:** 10.1097/MD.0000000000010385

**Published:** 2018-04-20

**Authors:** Yan Li, Ming-xi Zhu, Si-hua Qi

**Affiliations:** a Department of Anesthesia, The 4th Affiliated Hospital of Harbin Medical University; b Key Laboratory of Hepatosplenic Surgery, Ministry of Education, Department of General Surgery, The First Affiliated Hospital of Harbin Medical University, Harbin, China.

**Keywords:** cancer-specific survival, Epidemiology and End Results (SEER) database, marital status, overall survival, renal cell carcinoma, surveillance

## Abstract

Previous studies have shown that marital status is an independent prognostic factor for survival in several types of cancer. In this study, we investigated the effects of marital status on survival outcomes among renal cell carcinoma (RCC) patients.

We identified patients diagnosed with RCC between 1973 and 2013 from the Surveillance, Epidemiology and End Results (SEER) database. Kaplan–Meier analysis and Cox regression were used to identify the effects of marital status on overall survival (OS) and cancer-specific survival (CSS).

We enrolled 97,662 eligible RCC patients, including 64,884 married patients, and 32,778 unmarried (9831 divorced/separated, 9692 widowed, and 13,255 single) patients at diagnosis. The 5-year OS and CSS rates of the married, separated/divorced, widowed, and single patients were 73.7%, 69.5%, 58.3%, and 73.2% (OS), and 82.2%, 80.7%, 75.7%, and 83.3% (CSS), respectively. Multivariate Cox regression showed that, compared with married patients, widowed individuals showed poorer OS (hazard ratio, 1.419; 95% confidence interval, 1.370–1.469) and CSS (hazard ratio, 1.210; 95% confidence interval, 1.144–1.279). Stratified analyses and multivariate Cox regression showed that, in the insured and uninsured groups, married patients had better survival outcomes while widowed patients suffered worse OS outcomes; however, this trend was not significant for CSS.

In RCC patients, married patients had better survival outcomes while widowed patients tended to suffer worse survival outcomes in terms of both OS and CSS.

## 1 Introduction

Renal cell carcinoma (RCC) is the third-most lethal genitourinary malignancy, making it the seventh most common cancer (335,000 new cases, 2.4% of the total cases worldwide). There are approximately 693,000 deaths due to RCC worldwide per year.^[1]^ The survival of RCC is affected by many factors, such as the TNM stage, Fuhrman nuclear grade, RCC subtype, tumor size, molecular pathogenesis, treatment regimen, and socioeconomic status.^[2–4]^ Further, marriage, another important prognostic factor, has been reported to have a protective effect on the prognosis of many cancers.^[5–9]^ However, other researches have shown that marriage has mixed or no effect on survival among some cancer patient.^[10–13]^ Besides, these studies did not take both overall survival (OS) and cancer-specific survival (CSS) into consideration. Meanwhile, social network and social economic factors have significantly changed in the United States in the past decades. Two previous studies have implicated that marriage status might influence the survival of RCC,^[14,15]^ however, the results remain inconclusive. Therefore, in the present study, we used the Surveillance, Epidemiology, and End Results (SEER) database to explore the impact of marital status on survival outcomes of renal cancer patients.

## 2 Material and methods

### 2.1 Patients

This study retrospectively analyzed the SEER database, reported by the US National Cancer Institute, which collects patient demographics and publishes cancer incidence and survival data from 18 population-based cancer registries. The dataset used for the analysis was documentation Version April 2016, which covers approximately 30% of the US population. The SEER database is openly accessible and freely available for researchers. We obtained the research data files with the user number 12948-Nov2015. Because the data extracted from this database were anonymized and de-identified before being released, our research did not require patient informed consent.

**Figure 1:**
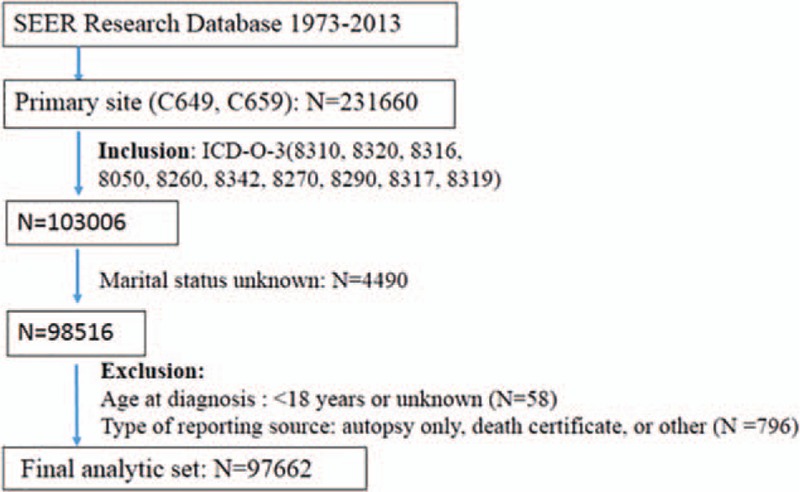
The subject selection algorithm. Note that some patients met >1 exclusion criteria. SEER indicates Surveillance, Epidemiology, and End Results. SEER =  Surveillance, Epidemiology, and End Results.

Individuals with renal tumors were analyzed based on the “kidney and renal pelvis” as the primary site, according to the International Classification of Diseases for Oncology, Third Edition (ICD-O-3) codes C64.9 and C65.9. All these patients were diagnosed between 1973 and 2013. Only patients with the main histologic subtypes (clear cell, papillary, chromophobe, and collecting duct) were included. Histologic confirmation of RCC was determined using the following *ICD-O-3* codes: clear cell (8310, 8320, 8316), papillary (8050, 8260, 8342), chromophobe (8270, 8290, 8317), and collecting duct (8319). We categorized marital status into married and unmarried. The unmarried group included divorced/separated, widowed, and single subgroups. The patients were moreover divided into “insured” and “uninsured” groups according to their insurance status. The exclusion criteria included patients with unknown marital status, age at diagnosis of <18 years or unknown age, and all autopsy or death certificate cases. This method resulted in a total of 97,662 RCC patients being included in the analysis. The subject selection algorithm is shown in Figure 1.

### 2.2 Statistical analysis

Two endpoints, OS and CSS, were used in this study. In the OS analysis, any cause of death was considered an event and survivors were considered as censored events. In the CSS analysis, death due to RCC was considered an event and deaths of other causes or survivors were considered as censored events. We used χ^2^ tests to analyze the baseline characteristics of the patients in the 4 marital subgroups. The Kaplan–Meier method with the log-rank test was used to analyze the effects of each factor on CSS and OS. We also used univariate and multivariate Cox's regression models to estimate the hazard ratio (HR) and exact 95% confidence interval (CI). All statistical tests were performed using SPSS 23 software (Chicago, IL). Finally, all tests were 2 sided, and the significance level was set at *P* < .05.

## 3 Results

### 3.1 Patient baseline characteristics

A total of 97,662 eligible RCC patients were finally enrolled during the study period from 1973 to 2013 in the SEER database. Of these, 64,884 (66.5%) patients were married at the time of diagnosis and 32,778 (33.5%) were unmarried (including 9831 (10.1%) divorced/separated, 9692 (9.9%) widowed, and 13,255 (13.5%) single). The 4 subgroups (married, divorced/separated, widowed, and single) were compared. Details of the patient demographics and pathological features are shown in Table 1. Of the total patients, 62,044 (63.5%) were males and 35,618 (36.5%) were females, suggesting a higher risk of RCC in men. There were significant differences in demographics and pathological characteristics, including sex, age, ethnicity, grade, insurance status, histological type, SEER stage, and therapies between the married and unmarried groups. Compared with other marital statuses, among the widowed group, the proportions of women (71.0%) and older individuals (80.7%) were the highest *(P* *<* .001*)*. In addition, the likelihoods of localized tumors (68.9%) and surgical treatment (10.7%; partial nephrectomy or total/radical nephrectomy) were lower among widowed patients than for the other groups. Single patients were less likely to be female and were much younger (≤65 years).

**Table 1:**
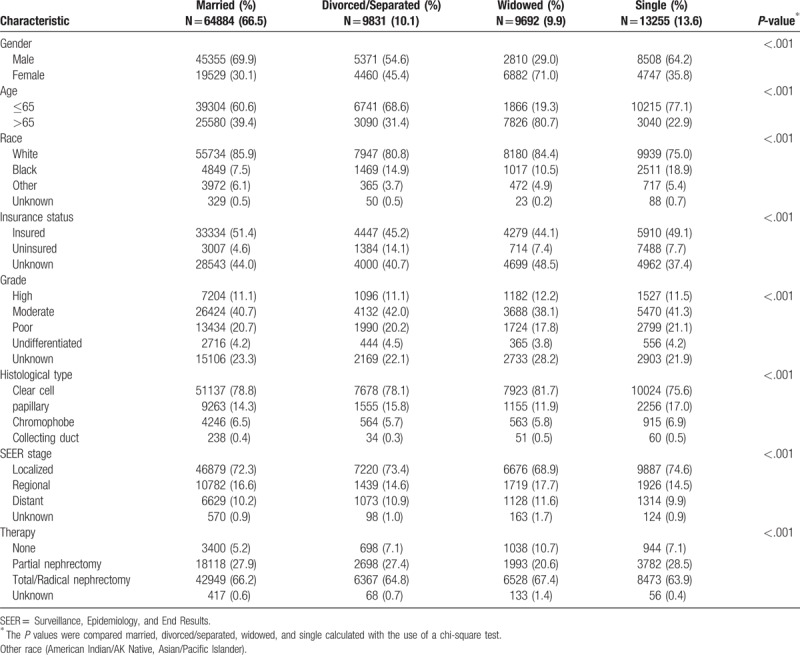
Patient baseline demographic and clinical characteristics.

### 3.2 Effects of demographics and pathological features on overall and cancer-specific survival in the SEER database

**Figure 2:**
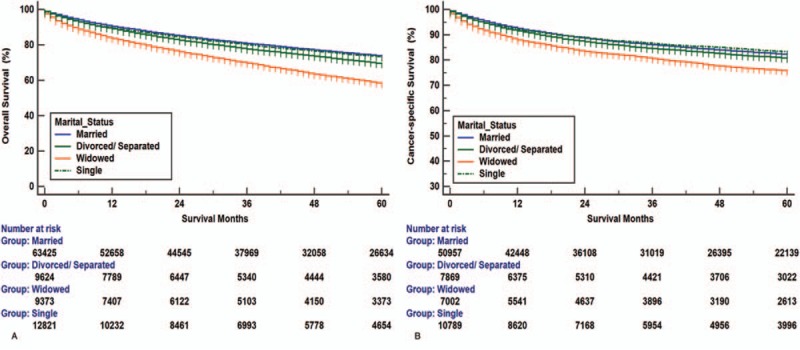
Overall survival and cancer-specific survival curves of renal cancer patients according to marital status. (A) Overall survival of renal cancer patients according to marital status, log-rank χ^2^ test = 1495.760, *P* *<* .001. (B) Cancer-specific survival curves of renal cancer patients according to marital status, log-rank χ^2^ test = 242.6960, *P* *<* .001.

In this study, we used Kaplan–Meier analysis to calculate the OS and CSS of RCC patients (Table 2). The 5-year OS rate of the married group was 73.7%, while those of the separated/divorced, widowed, and single groups were 69.5%, 58.3%, and 73.2%, respectively. The survival outcome among the 4 marital subgroups was significantly different (*P* < .001) (Fig. 2A). The 5-year CSS rate of the married group was 82.2%, while those of the separated/divorced, widowed, and single groups were 80.7%, 75.7%, and 83.3%, respectively (*P* < .001) (Fig. 2B). Widowed patients had the lowest rate of survival and the shortest survival time (*P* < .001) (Fig. 2). Besides marital status, sex, ethnicity, age, tumor grade, insurance status, histological type, SEER stage, and therapy methods were also shown to be significantly associated with OS and CSS by Kaplan–Meier analysis (Table 2). The 5-year OS and CSS rates of the male patients were both lower than those for the female patients (70.3% vs 74.0%; 80.7% vs 83.3%).

**Table 2:**
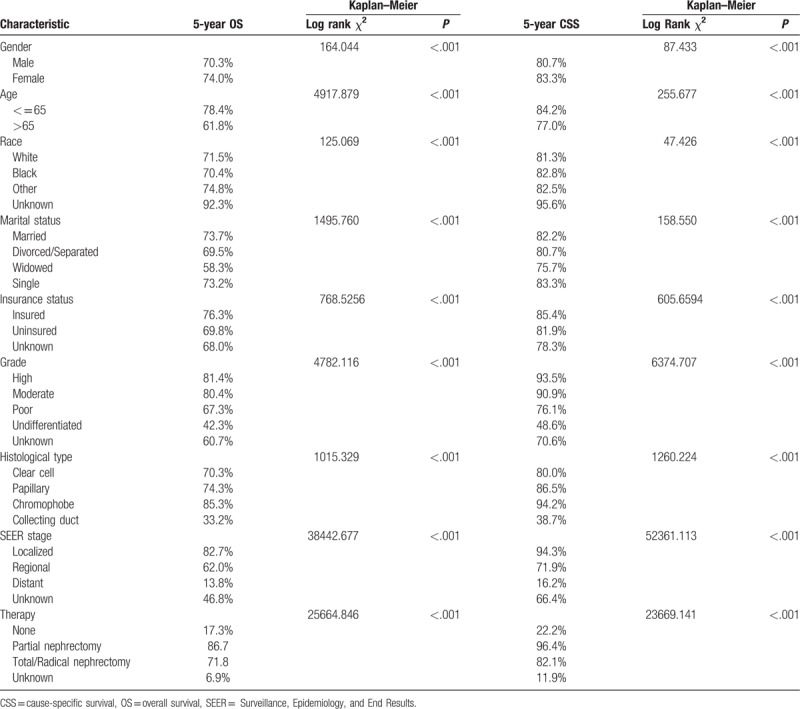
Kaplan–Meier analysis overall survival and cancer-specific survival for renal cell carcinoma.

### 3.3 Identification of prognostic factors for patients with RCC

To identify the prognostic factors for patients with RCC, we further performed univariate and multivariate Cox analyses of the above-mentioned prognostic factors (Tables 3 and 4). Characteristics including age ≥65, divorced/separated, widowed, uninsured, poor/undifferentiated, collecting duct histology, and regional/distant metastasis were considered as independent poor prognostic factors for OS and CSS in patients with RCC. For OS, multivariate Cox analysis indicated that, compared to married patients, separated/divorced (HR, 1.237; 95% CI, 1.192–1.285; *P* *<* .001), widowed (HR, 1.419; 95% CI, 1.370–1.469; *P* *<* .001), and single patients (HR, 1.178; 95% CI, 1.136–1.221; *P* *<* .001) were independent prognostic factors for poor survival (Table 3). In term of CSS, multivariate Cox analysis similarly showed that marriage was a protective factor for RCC prognosis (separated/divorced: HR, 1.105; 95% CI, 1.045–1.168; *P* *<* .001; widowed: HR, 1.210; 95% CI, 1.144–1.279; *P* *<* .001; single: HR, 1.089; 95% CI, 1.034–1.048; *P* *=* .001) (Table 4).

**Table 3:**
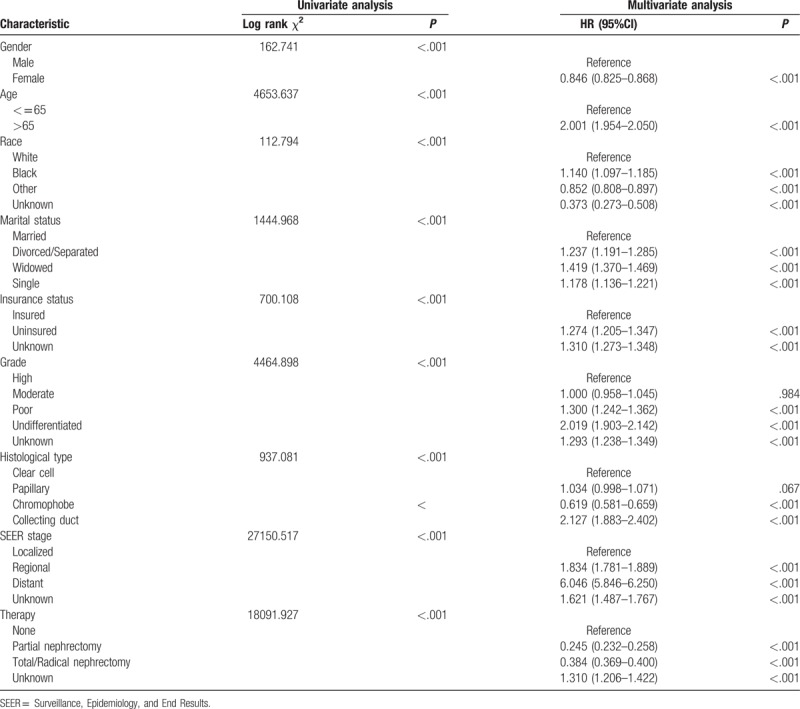
Univariate and multivariate analysis of renal cell carcinoma overall survival.

**Table 4:**
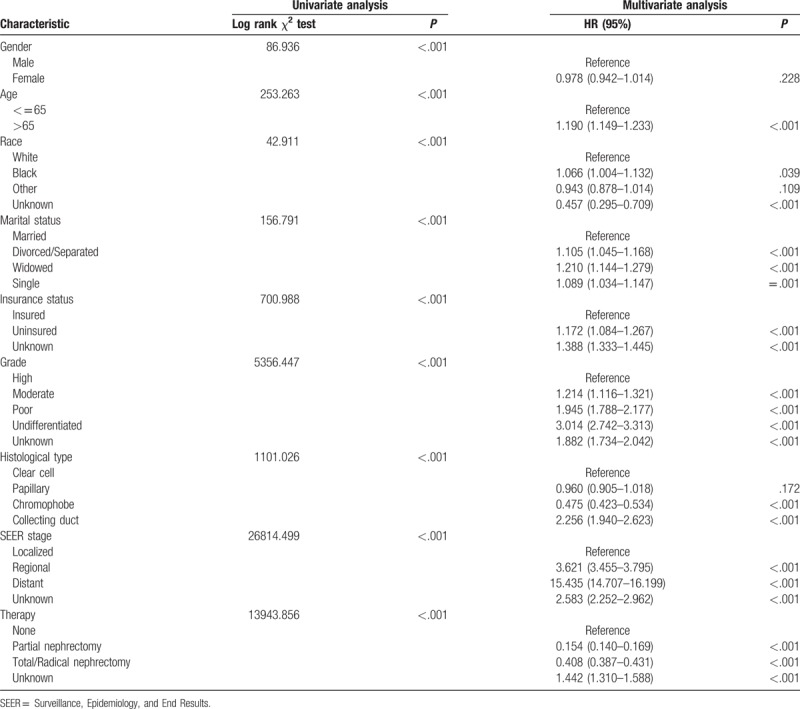
Univariate and multivariate analysis of renal cell carcinoma cause specific survival.

### 3.4 Stratified analyses of the impact of marital status on overall and cancer-specific survival based on insurance status

**Figure 3:**
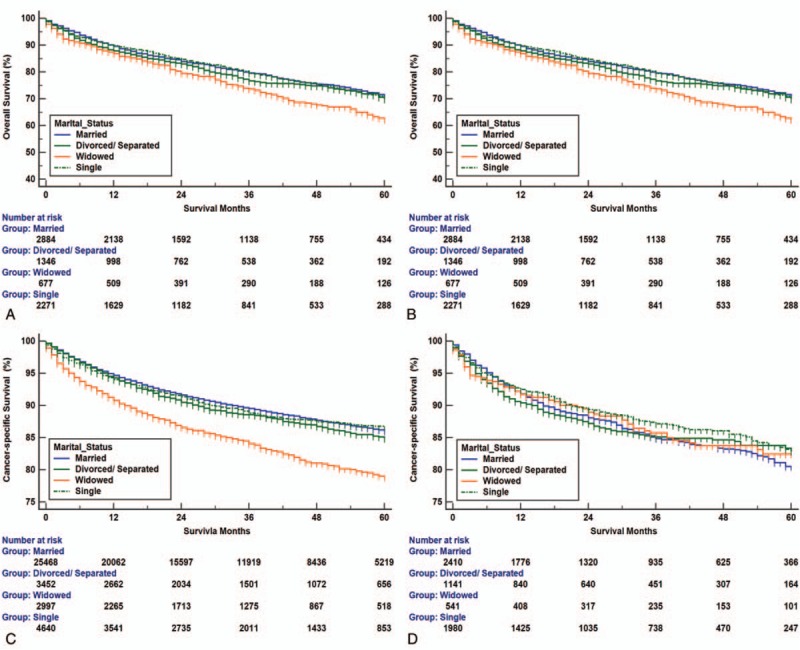
Overall survival and cancer-specific survival curves of renal cancer patients according to marital status in insured and uninsured patients. (A) Overall survival of renal cancer patients according to marital status in insured patients, log-rank χ^2^ test = 423.532, *P* *<* .001. (B) Overall survival of renal cancer patients according to marital status in uninsured patients, log-rank χ^2^ test = 22.036, *P* *<* .001. (C) Cancer-specific survival curves of renal cancer patients according to marital status in insured patients, log-rank χ^2^ test = 94.63, *P* *<* .001. (D) Cancer-specific survival curves of renal cancer patients according to marital status in uninsured patients, log-rank χ^2^ test = 2.958, *P* = .398.

To assess the potential reasons for the survival disparity between married and unmarried patients, we further explored the effect of insurance on survival. We used Kaplan–Meier analysis to calculate the OS and CSS of RCC patients in the insured and uninsured subgroups (Fig. 3). For patients who were covered by insurance, widowed patients had a significantly worse median survival and 5-year OS than the married, divorced/separated, and single patients (median survival: 69.7, 67.2, 59.9, and 69.4 months; 5-year OS rate: 77.9%, 74.0%, 62.8%, and 78.4%, respectively). Widowed patients also had a significantly worse median survival and 5-year CCS than the other patients (median survival: 74.3, 73.6, 69.6, and 74.2 months; 5-year CSS rate: 86.0%, 84.9%, 78.9%, and 86.5%, respectively). For uninsured patients, widowed patients had a significantly worse median survival and 5-year OS than the married, divorced/separated, and single patients (median survival: 65.6, 63.7, 59.4, and 65.2 months; 5-year OS rate: 71.1%, 69.9%, 62.3%, and 70.7%, respectively). However, no significant difference was found for CSS (median survival: 71.1, 70.8, 71.3, and 72.4 months; 5-year CSS rate: 80.5%, 83.4%, 82.5%, and 82.9%, respectively; *P* = .3982). Finally, we used multivariate Cox regression to analyze the effects of marital status on OS and CSS according to insurance status (Table 5). The results showed that, in both the insured and uninsured subgroups, compared with the married patients, the unmarried patients had worse OS outcome. However, no difference was found in CCS in both subgroups.

**Table 5:**
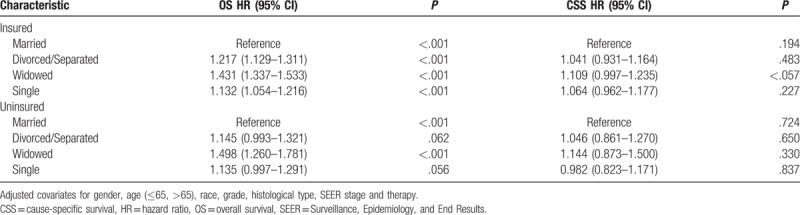
Multivariate analysis of marital status on renal cell carcinoma overall and cause-specific survival according to different insurance status.

## 4 Discussion

Our study showed that married patients enjoyed both better 5-year OS and CSS outcomes than unmarried patients, including divorced/separated, widowed, and single patients. In addition, widowed patients had the lowest 5-year OS and CSS rates compared with other patients. In this study, we also found that, after adjusting for sex, age, ethnicity, tumor grade, insurance status, histological type, SEER stage, and therapy methods, multivariable Cox analyses showed that married patients enjoyed both better OS and CSS outcomes than all subgroups of unmarried patients. Stratified analyses showed that, in both the insured and uninsured groups, married patients had better survival outcomes while widowed patients suffered worse OS outcomes; however, this trend was not significant for CSS.

In addition, we also found that RCC tended to occur more frequently in males and that male sex may be associated with poor prognosis, which was in accordance with 2 previous researches.^[16,17]^ Patients with collecting duct histological type also had lower OS and CSS, possibly due to the frequent presence of metastasis (>70% of patients) at diagnosis.^[18]^

Interestingly, some studies have suggested that uninsured status is associated with poor prognosis in certain cancers.^[19]^ In our study, this trend could also be observed, since the percentage of patients with insurance was the lowest in the widowed group, at 44.1% (compared with 51.4%, 45.2%, and 49.1% in the married, divorced/separated, and single groups, respectively). Compared with married people, unmarried patients had a lower proportion of surgical treatment, which may be partly related to their survival disadvantage. The reason for this finding may be that unmarried patients lack the needed support from a spouse, have unhealthy lifestyles, and lack financial resources. On the other hand, married patients can receive support from their spouses, such as receiving medical assistance, assisting in activities of daily living, and medication reminders.^[20]^ Lifestyles may also be affected by marriage; for example, divorced individuals and their offspring have been reported to more frequently suffer from alcohol and tobacco consumption, drug abuse, and sexual problems.^[21]^ Additionally, compared with unmarried persons, married people may have stronger financial resources,^[19,22]^ which leads to early detection and timely medical care. Our findings also suggest that marriage may have a protective effect on cancer patients. The difference in survival among patients with different marital statuses may depend, at least partly, on the possibility of better access to medical remedies. Another important factor, psychological support, may contribute to the better prognosis among married patients. When people are diagnosed with cancer, many may suffer from psychological distress, anxiety, and depression.^[23]^ Several studies have reported that married patients can share the emotional burden with their spouses, resulting in survival benefits.^[1,24,25]^ The potential mechanisms underlying this correlation might be explained by the fact that the immune and endocrine functions are disturbed by psychological distress.^[26–28]^

Though our present study is not the first to analyze the survival disparity between married and unmarried RCC patients, we did not simply replicate these previous studies. Actually, we made a further analysis to seek out possible explanations for the survival difference. In both previous studies,^[14,15]^ insurance status was not included as a covariate, and the effect of insurance on the survival outcome of patients with renal cancer was not considered. Herein, we used SEER data to elucidate whether insurance status, in addition to ethnicity, age, and SEER stage, among other factors, may play an important role in the survival disparity. Besides, in Wang et al's report,^[14]^ a total of 62,405 eligible patients diagnosed between 2004 and 2013 were enrolled; however, in our study, an even bigger sample of 97,662 eligible patients diagnosed between 1973 and 2013 were included. Additionally, in Miao et al's report,^[15]^ the kidney cancer patients included both renal cancer and renal pelvis cancer patients; the pathology of these 2 types of cancer is completely different. However, in our present study, we only enrolled RCC patients.

In this present study, some restrictions must be interpreted with caution. First, all types of therapy for RCC could not be provided by the SEER database, which may influence the association between marriage status and prognosis. Besides, other factors of improving prognosis, such as health behavior variables and socioeconomic status, were also not recorded. Second, the duration and satisfaction of marriage may impact the effect of marital status. However, the SEER database only provides the marital status at diagnosis. Third, as a retrospectively study, we could not avoid various forms of missing data (such as marital status unknown), which might be liable to inaccurate conclusions.

Despite the stated limitations, this study demonstrated that marital status affects both OS and CSS in RCC patients, with data from the large population-based SEER database. Our study suggests that married patients have better survival outcomes than unmarried patients after adjusting for known confounders. Especially, our study also showed that widowed patients are at higher risk compared with married patients in terms of poor OS and CSS; this disadvantage was particularly striking in insured patients. Taken together, this study highlights the importance of psychological intervention for cancer patients during treatment, especially for those who are unmarried.

## Author contributions

**Formal analysis:** Ming-xi Zhu.

**Writing – original draft:** Yan Li.

**Writing – review & editing:** Sihua QI.
